# Hospital Reports

**Published:** 1902-11-01

**Authors:** 


HOSPITAL REPORTS.

The Secretary of the General Hospital, Birmingham writes: Your reviewer has again done an injustice to this hospital in his comment in the last three lines of the paragraph on page 71. I may remind you that I previously pointed out that for some few years the statistical report of diseases and operations is published separately in order that the annual report may be issued to the governors before the annual meeting, which is held in the middle of March.

*** In our notice of the General Hospital, Birmingham, we omitted to state that the medical statistics of this hospital are published as a separate report.—Ed. T. H.

